# Preparing allied health students for placement: a contrast of learning modalities for foundational skill development

**DOI:** 10.1186/s12909-023-04086-7

**Published:** 2023-03-15

**Authors:** Laura Rossiter, Ruth Turk, Belinda Judd, Jennie Brentnall, Chloe Grimmett, Emma Cowley, Keith McCormick, Deborah Thackray

**Affiliations:** 1 School of Health Sciences, University of Southampton, Southampton, United Kingdom; 2 Faculty of Medicine and Health, University of Sydney, Sydney, Australia

**Keywords:** Simulation-based education, Online learning, Clinical placement, Allied health students, Occupational therapy, Physiotherapy, Podiatry

## Abstract

**Background:**

With increasing pressure on placement capacity for allied health students, a need for novel and creative means through which students can develop foundational skills and prepare for practice-based learning opportunities has arisen. This study aimed to explore the experiences of domestic and international first-year students completing pre-clinical preparation programs, contrasting between in-person simulation and online options to contribute to best practice evidence for program design and delivery.

**Methods:**

First-year students from physiotherapy, podiatry and occupational therapy self-selected to either a one-weeklong in-person simulation program or an online preparation for placement program. An integrative mixed-methods approach was employed. Qualitative findings from student focus groups were analyzed by reflexive thematic analysis and complemented by quantitative pre-post questionnaires which were examined for patterns of findings.

**Results:**

There were 53 student participants in the study (simulation n = 29; online n = 24). Self-selecting, international students disproportionately opted for the simulation program while older students disproportionately selected the online program. Students appeared to benefit more from the simulation program than the online program, with alignment of focus group findings to the quantitative questionnaire data. The in-person simulation allowed students to *apply* their learning and practice patient communication. All simulation students reported asubsequent increase in confidence, although this seemed particularly marked for the international students. By contrast, the online program was most effective at developing students’ clinical reasoning and proficiency with documentation. Both programs faced minor challenges to student perceived relevance and skill development.

**Conclusion:**

Both online and in-person simulation preparation programs were perceived to enhance readiness and foundational skills development for novice allied health students, with the practical nature of simulation generating more advantageous findings. This study provides useful information on the benefits and challenges of both types of delivery for foundational skills development and/or clinical preparation of allied health students.

**Supplementary Information:**

The online version contains supplementary material available at 10.1186/s12909-023-04086-7.

## Background

Clinical practice experiences remain integral to allied health professions (AHP) pre-registration curricula. Accrediting bodies of many allied health professions in the UK, and of occupational therapy worldwide, mandate a minimum of 1,000 h of practice-based learning during training for professional qualification and registration [1, 2]. The current healthcare climate and the increasing demand for allied health placements necessitate exploring creative ways to expand placement capacity and enhance clinical experiences.

In responding to the challenges of preparing allied health students for, and maximizing their learning in clinical placements, it is essential to understand students’ challenges, preferences, confidence, and preparation with different learning modalities [3]. This knowledge can enable educators to develop strategies and programs that best meet learning objectives whilst considering stakeholder acceptability and preferences within resourcing and logistical constraints.

International students are an important and growing sub-group to consider, representing 24% of all higher education enrolments in the UK in 2021/22 [4]. Commencing practice education is particularly challenging for those international students from diverse cultures and for whom English is not the first language, such as the 22% of international students from China or 18% from India [4]. For these students, adapting to cultural differences and experiencing communication challenges in the transition to clinical practice can be particularly stressful [5]. Students without English as a first language have previously described challenges communicating confidently with service users, peers and educators and articulating themselves in healthcare settings [6]. Adopting contemporary ways of learning involving groupwork and simulation could also be challenging. Mitchell et al. [7] found that language acquisition and acculturation are important factors for successful learning, with ‘finding and expressing oneself’ as central themes. Practical difficulties associated with changes in environment, discrimination, loneliness and homesickness potentially creating further challenges. Communication education and immersion in local culture before placement can demonstrably improve students’ confidence and their patient interactions [8].

Simulation-based education aims to recreate aspects of real-life tasks, events, and experiences whilst allowing tailoring for learning. Through deliberate and repeated practice in a safe learning environment with feedback, students participating in simulation-based education acquire and refine clinical skills, knowledge, attitudes, and behaviors [9, 10]. Simulation supports learner-centered immersion, aids in the transition into clinical setting learning and can supplement or replace a portion of clinical placement hours [11–15]. Simulation is therefore increasingly utilized to enable health professional students to safely learn to transfer theoretical knowledge into practice and may provide a safe psychological space for international students to overcome some of the challenges and barriers they face and create positive initial clinical experiences. In-person simulation programs, however, can be costly, resource-intensive, and reliant on safe, close physical proximity of students and providers which has not always been feasible in the pandemic climate. Accordingly, there is increased attention on what might be attained through dynamic and reflexive learning modalities that can be delivered online.

Online education consists of learning activities whereby students are physically distant from educators requiring technology-facilitated delivery methods [16]. Online learning may encourage student-centered learning that is easily manageable and accessible to students [17]. An online program that allows students to develop foundational skills when in-person classes are not feasible is an attractive proposition. However, literature is conflicting regarding the efficacy and acceptability of online learning, and there is scant literature concerning online learning specifically for clinical preparation in allied health students [18, 19]. The acceptability of online learning, its potential contribution to the clinical preparation of allied health students from varied backgrounds, and its efficacy for foundational skill development compared to in-person simulation-based education requires further exploration.

The study aimed, therefore, to explore and contrast students’ experiences and their development of foundational clinical skills in a one-week intensive in-person simulation program compared to an online learning program. Both programs were designed with the same learning objectives: to prepare allied health students for early clinical placement experiences. This study specifically sought to address the following research questions:

1. What are the benefits and challenges from a student perspective of adopting in-person simulation compared to online learning for foundational clinical skills development and placement preparation?
2. How does students’ perceived readiness for placement differ between in-person simulation and online learning?

## Methods

To address the aim and research questions, this study employed a mixed model research design seeking complementarity among approaches used concurrently [20]. Rich focus group discussions (qualitative) paralleled descriptive comparisons through pre- and post-intervention questionnaires (quantitative). The study was approved by the host university’s Human Research Ethics Committee (Approval No. 64,382) and voluntary, informed consent was gained from all participants.

### Participants

Study participants were recruited from the first-year student cohorts in occupational therapy, physiotherapy, and podiatry in the 2020–2021 academic year (n = 133). Students were eligible for participation if they were due to undertake their first clinical placement in June 2021 and were available for participation in one of the programs (simulation versus online) in the week before that placement.

General program information was provided to course cohorts during classes. Eligible students received formal study information and a consent form by email from a research team member not associated with their course (LR). Participants indicated their program preference when giving consent. Participants could opt-in to the simulation program, which had a maximum capacity of 30 students, or the online program. For learning equity, all first-year students could access the online learning program irrespective of participation in the research study.

Study participation involved the completion of pre-post questionnaires and completion of the one-week placement preparation program (simulation or online), for which students were offered a £15 voucher in recognition of their participation. All students were also invited to participate in a focus group, though participation was still recognized if they did not attend this component. They were free to withdraw from the study at any time.

### Intervention

Both the simulation and online programs addressed generic learning outcomes in the following areas: placement supervisory processes, personal learning goals, communication skills, professional behavior, foundation patient assessment, clinical reasoning skills leading to basic interventions, and reflective practice. Each program is detailed below.

#### Simulation Program

The week-long simulation (sim) program was designed and facilitated by the host institution’s allied health education staff (facilitators) with experienced clinical educators. The program was based on the conceptual framework by Chu et al. [21] and the experience of designing multi-disciplinary simulation programs at the collaborating university (BJ and JB). Replicating clinical placement features, the week commenced with program and learning outcome orientation, including interprofessional discussion of expected behaviors and identification of individual learning goals. Patient interactions were simulated throughout the week and utilized experienced simulated patients, who were supported by the facilitators and clinical educators. Introductory sessions were ‘fishbowl’ simulation scenarios where students took turns leading parts of the patient interaction while peers observed. These scenarios focused on patient-centered communication skills conducted with the interprofessional group.

Over the next three days, students also worked with facilitators and clinical educators in small, profession-specific groups, participating in simulations of interactions relevant to their profession. Each session had three phases: pre-briefing, active participation in a simulation scenario with turn-taking and debriefing with the simulated patient. There were three interprofessional patient scenarios, each developed with input from all three professions and clinical educators to ensure relevance of the profession as well as generic learning outcomes. The simulations also included a caregiver or relative to add further realism to the scenario and encourage additional communication skill development. Each professional student group rotated through the scenarios across the three days. On the final day, students were assigned to one of three interdisciplinary meetings to review a patient scenario encountered throughout the week. Finally, students were invited to engage in a final reflection of the program and evaluation of learning outcomes.

#### Online program

The online learning program was designed by a member of the host university academic staff (DT) with experience in instructional design. The content comprised a suite of resources that were then collated and presented on an intranet site by the first author. The materials covered varying topics to prepare students for placement, such as patient-centered communication skills, introductions to different placement settings, and placement supervisory approaches. Interactive materials included 360° images of three different placement settings (acute ward, outpatients and home setting) that students could virtually navigate with pop-up content to help them familiarize themselves with each setting. Students were encouraged to answer case study questions on the patients likely to be seen in each setting. The online materials, therefore, had similar features to the simulation program but without the practice, feedback and debriefing, or the structured peer interaction the simulation program provided.

### Data collection tools

#### Demographic data

Demographic data were collected from each participant on study registration. These included profession, gender, previous clinical experience, cultural identity, and enrolment category (domestic/international).

#### Focus groups

Three focus groups were conducted via online videoconferencing: two for simulation program participants (one each for domestic students and international students), and one for the online program participants. The interview questions for each focus group were similar and centered around the students’ perceptions of their preparedness for placement from their experiences of the programs, as well as challenges encountered and recommendations for program improvements. The focus groups were conducted within two weeks of program completion and then transcribed from the video inclusive of significant non-verbal cues (nods etc.).

#### Pre-post questionnaire

A brief survey completed before and after the programs asked students to rate their confidence in 17 skills relevant to a first placement experience (see Additional File 1) using a 5-point Likert scale: strongly disagree, disagree, undecided, agree, strongly agree. This survey was previously used in an internal evaluation of a discipline-specific simulation program at the collaborating university. Students completed the questionnaires in an online form.

### Data Analysis

Focus group data were analyzed using a reflexive thematic approach as outlined by Braun and Clarke [22, 23]. The data were initially coded by the first author (LR) in relation to the research question. LR, although involved in health professional education, had not previously had any simulation experience and did not conceptualize the programs, providing a degree of independence felt to be important during data analysis. The assigned codes were then reviewed, discussed, and clarified by the co-authors to ensure codes were interprofessionally relevant and reflected the diverse experiences of authors. A second co-author (CG), who has a background in qualitative research but not in allied health professions education, then used the agreed codes to code one of the focus groups to ensure the rigor of the analysis approach. Differences between the two coders were discussed and resolved, and themes were defined.

The questionnaire data were analyzed descriptively by question, given the nature of the questions and response scales, the unequal group sizes, and the small number of simulation participants. Patterns in the top and bottom rankings were considered alongside the dominant qualitative themes in a mixed analysis.

## Results

There were 53 study participants. A self-selected group of 29 students completed the in-person program and participated in the research (simulation group). The online learning was available to all students across the three professions (n = 133), of which 24 who had not participated in simulation consented to participation in the research (online group). All participants in the online group and most (86%) in the simulation group were female (see Table 1). Most participants (69% of the simulation group and 54% of the online group) were studying occupational therapy.

**Table 1:** Participant characteristics

Characteristic	SIMULATION (n = 29)	Online (n = 24)
**Gender**		
Female	25	24
Male	4	0
**Age**		
18–23	24	12
24–29	4	6
30–35	0	2
36–41	0	1
42–47	1	2
48–53	0	1
**Ethnicity**		
White or Caucasian	16	21
Black or Black British African	0	1
Asian or Asian British	12	2
Any other Ethnic Group	1	0
**Course Program**		
Occupational Therapy	20	13
Physiotherapy	6	9
Podiatry	3	2
**Program**		
Domestic	18	22
International	13	3
**Clinical Experience**		
None	13	7
< 1 week	8	4
1 week – 1 month	4	6
1 month – 3 months	1	0
3 months – 6 months	0	1
> 6 months	3	6

Most (11 of 13) international students were from Hong Kong and chose the simulation group. Conversely, most older students were in the online group, where one-quarter were aged over 30 years, compared with only one student in the simulation group. Students in the online group also reported more prior clinical experience, with 54% reporting more than one week of experience compared to 28% in the simulation group.

Twelve of the 29 simulation students participated across the two simulation focus groups, discussing both programs but with a focus on the simulation program. The international student group included one male and four females from occupational therapy and physiotherapy, while the domestic student group included two males and five females across all disciplines. Four of the 24 online students participated in the online program-specific focus group; two occupational therapy students and two physiotherapy students. Accordingly, in the qualitative findings reported below, focus group participants numbered 1–12 were from the simulation group, and 13–16 were from the online group. When attributing comments, ‘Int’ denotes an international student, and ‘dom’ denotes a domestic/home student. All students in the online group have the attribution ‘onl’ to distinguish them since all were domestic students. Occupational therapy is abbreviated to OT, podiatry to Pod and physiotherapy to PT. Consistent with the mixed-methods study approach, qualitative findings are presented thematically. The quantitative findings are presented alongside the skill and personal development theme with which they best align.

## Qualitative findings

**Fig. 1:**
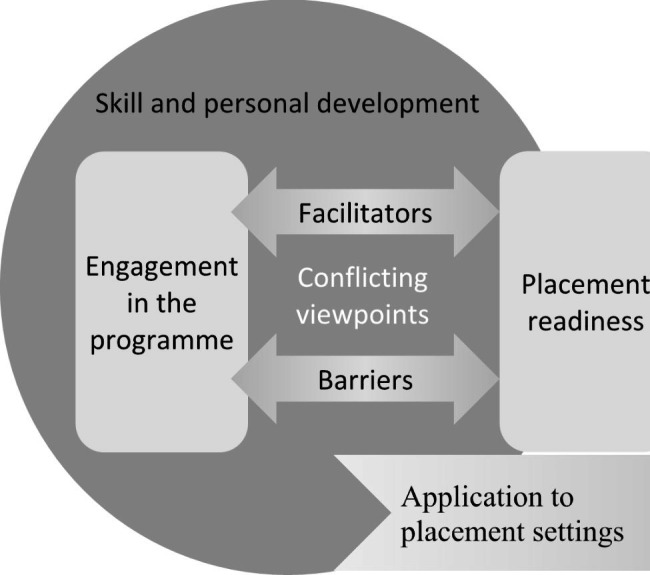
Relationship between Qualitative Themes

Seven inter-related themes were identified from the focus group data (see Fig. 1 for an illustration of the relationships between these themes). Skill and personal development was the dominant theme, and participants discussed development specifically in relation to application in their placement settings. Skill development was supported by engagement in the respective programs and in turn related to perceptions of placement readiness that carried through into placement settings. Participants shared factors within the programs that acted as facilitators and barriers to engagement in the program and thereby their perceived placement readiness. In some examples, student viewpoints regarding facilitators and barriers were conflicting.

### 1. Skill and personal development

Overall, skill and personal development was raised as the most important benefit of participating in the programs. The nature of the development varied between the two programs, with the students in the simulation program speaking more to their confidence in their skills and personal development than the students in the online program.

Specifically, simulation students felt better equipped to communicate with their clinical educator on placement and more able to communicate with service users, especially using techniques to build rapport and understanding *‘how you approach each individual differently and get to know the patient.’* (4-Dom-OT). The international students found it particularly useful to improve their phrasing.

*I really learnt how to avoid or not use specific language and just simple words. .. that can really ease the patient’s mind* (11-Int-PT)

In contrast the online students did not feel that their program improved their verbal communication because *‘it wasn’t really tested’* (15-Onl-OT).

Simulation students reported that specific clinical (or professional) skills and knowledge were developed throughout the simulation program. This included practice conducting assessments and understanding how the roles of different members of the multidisciplinary team differ. The development of specific clinical skills was not commented on by students completing the online program.

All simulation program students felt that their confidence improved, and some found positive feedback particularly reassuring.

*Some people are usually quite critical of themselves. .. so a lot of people were quite surprised when they were complimented for what they were doing* (6-Dom-OT)

Both domestic and international simulation students believed participating pushed them out of their ‘comfort zone’ (1-Dom-Pod). Some international students expressed anxiety beforehand but noticed a change in how they felt about speaking in front of the group as the week progressed.

*In the first day it was like don’t pick me. .. and then in the last days it was like I’ll go, I don’t mind’ (smiles)* (8-Int-OT)

Simulation students overwhelmingly felt the process was transformative. A student explained that they would have been *‘completely different’* if they had not completed the week (5-Dom-PT). Another thought that they would have been *‘very stressed’* (9-Int-OT). Contrastingly, all online students felt the online program did not significantly improve their confidence as they were unable to apply their learning.

*‘It’s hard to answer when you haven’t done it yet to know how confident you feel. .. there was just no way of measuring’* (13-Onl-OT)

The survey results concurred with these findings regarding skill and personal development. Increases in overall preparedness post-program compared to pre-program were higher for simulation students (89%) compared to online students (42%), although students who selected simulation rated their overall preparedness lower before the programs began. The areas that the highest and lowest proportions of students in each program rated as improving by at least one category are shown in Table 2. For simulation students, the areas most frequently rated as improved primarily involved interpersonal and communication skills where students reported lower confidence before the program. Areas concerning conducting assessments, note taking and implementing interventions were rated highest for online students. Recognizing limitations and infection control were least frequently rated as improved by students in both programs.

**Table 2:** Areas students most and least frequently rated as improved after the program

Simulation Program	Online Program
**Areas most frequently rated as improved post-program**	
Adapting communication (65%)	Completing documents to legal requirements (71%)
Establishing rapport (62%)	Identifying key problems (67%)
Implementing interventions (62%)	Implementing interventions (67%)
**Areas least frequently rated as improved post-program**	
Recognizing limitations (28%)	Recognizing limitations (21%)
Manual handling (31%)	Explaining role (25%)
Infection control (31%)	Infection control (25%)

### 2. Application of learning in placement settings

Simulation students reported application of their learning in a placement setting where interactions with patients on placement and simulated patients in the simulated scenarios felt similar, with one student commenting that the scenarios were ‘exactly’ what they then experienced on clinical placement. Another saw their learning directly acknowledged:

*‘it helped my therapeutic use of self. .. my practice educator commented. .. she said I was really learning to use my personality more and I’m more aware of my body positioning. .. we got taught that when (student name) did her bit. .. she was told, oh you can pull the chair closer. .. those stuck with me that I took on to placement’* (8-Int-OT)

The online students rarely commented on applying their learning in a placement setting, with a student describing:

*‘if you are lucky you will retain it* [knowledge] *and then you can come back to it when you do get a chance to practice’* (2-Dom-PT).

A student reported implementing online learning without realizing their actions *‘actually linked back’* to what was included in the program. (16-Onl-PT). Clinical placement learning opportunities were more evident for simulation students.

### 3. Facilitators to engagement in the program

Typically, facilitator and clinical educator involvement enabled a useful and *‘immediate response’* (1-Dom-Pod) to simulation students’ questions and ensured university curricula content to be applied ‘*more smoothly’* (11-Int-PT). Students also felt their peers created a safe learning atmosphere where they were supportive and *‘willing each other on’* (4-Dom-OT), so that *‘making a mistake is not a problem’* (10-Int-OT).

Discussion of online program content across peers facilitated learning. Self-initiated daily meetings of occupational therapy students were used to work through the questions as a group. Both focus group participants from occupational therapy felt this was *‘really useful’* (15-Onl-OT) to *‘consolidate’* (13-Onl-OT) their knowledge, particularly drawing on peers’ relevant personal family experience. The physiotherapy students in the focus group were unaware of such interactions happening in their cohort but felt that participating in something similar would have been useful.

Online students overwhelmingly commented on the content of the case studies, finding them useful to understand the *‘depth of a person’* (16-Onl-PT) and to facilitate detailed discussion among the OT students who chose to work collaboratively. Additionally, all students had access to the online materials and content access was considered easy and *‘really clear and intuitive’* (13-Onl-OT).

All students reported that their chosen program was easy to engage with. Some online students commented that online learning was *‘familiar’* and *‘flexible’* when they *‘felt quite burnt out’* (14-Onl-PT). Whereas the simulation program was considered as a more *‘full experience’* (6-Dom-OT), and other students felt in-person learning would enable them to *‘turn up and get on with it’* (7-Dom-PT). For occupational therapy students specifically, simulation program selection was also motivated by simulation comprising part of their 1000 clinical hours and consideration of their limited campus teaching time throughout the pandemic.

### 4. Barriers to engagement in the program

Students from both programs felt that additional elements would have been helpful. For example, having goal setting activities more prominent at the start of the simulation week. Similarly, introductory content was considered *‘woolly’* (14-Onl-PT) by the online students, who also indicated that they would have liked case study answers provided to ensure they were *‘on the right track’* and encourage *‘extra research’* (13-Onl-OT).

Students also found elements of the simulation program introductory day unhelpful. International students did not find role-playing as a clinical educator useful as they lacked knowledge of clinical educator behavior, and the students also felt that the advice on initial email clinical educator contact redundant as they had made contact already.

Across both programs, elements not facilitating knowledge application were described as less conducive to participant engagement. Online students reported a general inability to get *‘hands on’* beyond applying written knowledge to the case studies. The introductory day was felt to be less interactive by the international simulation students, with one participant feeling that it *‘did drag’* (8-Int-OT). The days were described as long by all simulation students, with some reporting it left them *‘knackered’.* This contributed to their not accessing the supplementary online materials along with their knowledge that this content could be accessed on-demand. Processing a lot of information in another language was particularly exhausting for international students.

*‘My brain is so tired to process so many English’* (9-Int-OT).

Online students also mentioned that they were tired from the assessed work that preceded the program:

*‘I’ve then had to go onto a five-week placement. .. we’ve just come out of exams, we’re knackered’* (14-Onl-PT)

### 5. Facilitators to placement readiness

The online content was enjoyed by students from both programs. Online students described digital content in greater detail as this was their primary learning source. It was described as *‘innovative’* (15-Onl-OT) and *‘thought provoking’* (14-Onl-PT). An ability to ‘revisit’ the online content was a feature enjoyed by students from both programs, and the online students appreciated the program structure.

*‘I knew I had something to do each day like for motivation otherwise. .. I wouldn’t know where to start. .. I didn’t really know how to write a patient note [or] a discharge summary to a GP so I looked up how to do them and did them’* (13-Onl-OT)

Although a few students would have liked more guidance before taking their turn role playing with the simulated patient, the international students felt that the opportunity to be put on the spot improved their problem-solving:

*‘although the process is really tough. .. it forces us to really brainstorm instead of just spoon-feeding what we can do’* (9-Int-OT)

Practicing was appreciated by the simulation students, with the simulated patients felt to be convincing and the peer observation and feedback constructive. Clinical educator input also seemed to benefit most students, and they felt comfortable asking the educators questions. International students particularly valued facilitator feedback:

*‘the clinical educator’s feedback would be more convincing like I would trust their feedback and I would learn from their feedback’* (12-Int-OT)

### 6. Barriers to placement readiness

Simulation program elements that were less tailored to students’ placements were considered less conducive to enhancing their placement readiness. For example, *Common Placement Assessment Form* from the Chartered Society of Physiotherapy [24] was encouraged for all students reflecting on their progress, albeit the students found sections repetitive and some parts irrelevant to their simulation program experiences. For other students, not having their turn interacting with the simulated patient or their multidisciplinary team meeting allocation align with the case study most relevant to their placement was a barrier to transferring their learning. Congruent with their views about the difficulty of taking a turn and their lack of understanding of the clinical educator role, a few international students felt like someone acting as the clinical educator and leading the assessment should be included if the simulation program were to reflect the competencies expected of students in their first placement.

Similarly, some elements of online content were viewed as somewhat irrelevant to the placement setting. For example, a few online students felt some materials presented in connection with the acute care setting (e.g., Bristol stool or positioning charts) or particular 360° images were irrelevant for their upcoming placement. In this instance, these students worked through that content less thoroughly.

*‘I knew it wasn’t going to be relevant to my upcoming placement because I was in outpatients so I thought I’d rather not stuff anything else into my brain’* (14-Onl-PT)

### 7. Differing student viewpoints on facilitators and barriers

Finally, there were some topics where there was discrepancy between students as to whether they were facilitators or barriers to developing placement readiness. The optimal group size to facilitate learning was contentious, with all simulation students commenting that it was *‘valuable watching’* (3-Dom-OT) others but also that with more students in the group, there was less opportunity to participate. Although experiencing simulation in different group sizes, the domestic students thought that five students per group felt *‘most appropriate’* (6-Dom-OT). Online students generally found it more beneficial to work in larger groups.

The utility of the SOAP note guidance was not always obvious for simulation students. Some domestic simulation students found the resource useful, however, one student said that they did not use SOAP notes in their setting, and another commented that they left the program *‘even more confused’* (7-Dom-PT). A few simulation students and an online student felt that having a SOAP note example would have been helpful.

## Discussion

This study explored and contrasted students’ experiences of their development of foundational clinical skills in an immersive simulation program and an interactive online program in advance of their first clinical placement. Overall, students unanimously felt that both programs increased their perceived readiness for placement, although in different ways, highlighting the student experience of the characteristics of each learning modality. The simulation students reported that this program provided them with the opportunity and impetus to adapt their communication skills, establish rapport, and implement therapeutic interventions, thereby applying their classroom learning. International students reported entering into the program feeling particularly nervous but finished the simulation week feeling significantly more confident. The online students reported that their program increased their confidence in identifying key problems, implementing interventions theoretically and writing documents to legal requirements. These differences highlight how two programs with similar learning outcomes but with different features and levels of immersion offered different opportunities for skill development.

The diversity of students in the study was broadly reflective of the typical demographics of physiotherapy, occupational therapy, and podiatry cohorts at the host university. As a result, the students presented with the varied learning needs associated with their different personal characteristics and backgrounds (age, culture, prior education and clinical experience). Given the opportunity to select the program they perceived would best meet their learning needs, students with different characteristics chose each program. Choice is an important consideration in students’ engagement and commitment to learning [25], and these students reflected on factors such as their availability to commit and prior learning opportunities influencing their choices. Collectively, they demonstrated the potential of these contrasting programs to meet different learning needs and thereby provide accessibility for a range of students.

Whilst the learning outcomes were parallel in the two programs, the simulation program had an attendance commitment similar to placement to access the immersive and interactive learning opportunities. The realism of week’s program provided physical, emotional and conceptual fidelity that helped students to rehearse their professional behaviors within a psychologically ‘safe container for learning’ [26]. The stop-start approach in the interactions with simulated patients allowed students to make errors, be corrected, and then repeat the activity. The university staff facilitating the scenarios shared a strong understanding of the expectations of the first year of study, while clinical educators from local healthcare settings could link the experiential learning to contemporary clinical practice. This combination of supervision and support added to the sense of psychological safety, allowing the students to engage in interpersonal risk-taking [27]. Students also further reinforced this by providing each other with encouragement and respectful feedback, attaining the benefits of trust and peer learning that are valuable elements of simulation-based learning [28]. Together these simulation program features made for a successful learning experience in terms of students’ perceived gains in skills to complete simple assessments and interventions, and especially communication and rapport-building. These findings are consistent with the growing body of literature demonstrating growth in students’ self-efficacy following immersive simulation experiences [7, 13, 29, 30], illustrating the value of simulation in preparedness for placement [31].

The online program, in contrast, was continuously available, which enabled asynchronous and pulsed learning affording greater flexibility and accessibility than the simulation program. The inclusion of 360^o^ images of three clinical environments (acute, outpatients and a home setting), with case studies and supplementary materials linked to each setting, increased the level of rich description and demonstration for the learner. However, the less immersive case studies elicited less of an emotional connection than the in-person simulation. The cognitive dominance in the student learning is reflected in the students’ increases in confidence identifying problems and writing documents to a legal requirement. An interesting insight from the students was that the online program was attractive to those feeling tired from a challenging period of study and exams. The lack of observation, encouragement and correction from staff may have resulted in the online program being perceived as less challenging, which may have made for a more comfortable transition for some students.

Considering these overall features, the far greater uptake of the simulation program by international students within the study is striking. The benefits of a preparatory program perceived by international students, such as the opportunity to rehearse patient interactions and gain feedback to boost their confidence, are aligned with the simulation modality. This was evidently apparent to and motivating for the students at the point of program selection. It is important that programs that include culturally and linguistically diverse students are adequately designed to meet their needs. When it comes to learning in complex clinical environments, developing agile verbal and non-verbal communication whilst adapting to learning in a new culture may be a significant challenge [32]. Likewise, translating between back and forth between languages throughout the day may contribute to cognitive overload [33] and present a barrier to engaging in verbal reasoning and learning, resulting in tiredness and a disconnection from learning.

Culturally and linguistically diverse students may therefore require opportunities for cultural adjustment as well as developing communication skills, with learning enabled by grading complexity and providing positive experiences [34]. Cultural differences are an important consideration for educators who must manage the potential for simulation participants to experience stress and distress [35, 36]. In this study, international students found turn-taking was initially uncomfortable, but utilized initial peer observation to increase understanding before participation, and then utilised peer support throughout. Doing so they overcame their discomfort and increased their confidence to volunteer and engage in a valuable learning opportunity.

Students’ use and value of peer and social learning to facilitate personal and cognitive development [37] was evident in both programs. The simulation program was designed with students organized into small professional groups to support each other and learn through observation and participation in turn-taking. The online program was designed to be accessible for independent learning and did not facilitate formal peer learning. However, some students reported taking the initiative to form their own study peer support groups. Incidentally, by supporting one another, these students who collaborated in their respective programs informally enabled each other and learnt from one another. Peer learning can reduce anxiety and increase students’ confidence with clinical skills, problem solving, and critical thinking [38]. In-person peer collaboration and interaction were part of the design of the simulation program and this was reported explicitly as a significant learning facilitator.

As well as peers, the facilitators and clinical educators in the simulation program provided a level of support that enabled students to work through areas of discomfort. Encouraging students to try new roles with the support of peers throughout the simulations and debriefings likely enhanced the insights they gained from the, at times uncomfortable, process of reflecting on performance. The online program was designed without the opportunities for students to be encouraged to try activities outside of their comfort zones, and without supportive debriefing to aid reflection on any challenges encountered. It was recommended by online students that feedback or answers to the scenarios could have been provided. While this may have addressed gaps in knowledge and understanding, the inability to replicate the immersive nature of simulation, level of support, active reflection, and debriefing is a limitation of an online program that was designed to be accessible on demand and resulted in the limited opportunity for communication or behavior change.

Finally, when designing a program to enhance student readiness for placement, consideration must be given to ensuring constructive alignment with learning outcomes appropriate for the level of the learners and building upon their foundational knowledge [39, 40]. The design and content of both programs was appropriate for first year learners and both facilitators and clinical educators were aware of the knowledge levels of first-year students [41]. As the simulation scenarios played out, students were required to apply their knowledge to progress the scenario. Students reported feeling at times they did not have the required knowledge. However, they felt supported by having the clinical educator involved in the simulation as in clinical practice. Clinical educators therefore played an essential role in encouraging students to remain engaged and without fear of making errors as this was a safe learning environment. This suggests that students worked beyond their ‘zone of proximal development’ [42] with the encouragement from the educator. Students highlighted that by seeking confirmation as they would in a clinical placement setting from the clinical educator, it gave them the confidence to continue.

## Limitations

The modest sample sizes for each component of this study and investigation of the program implementation at one institution limit the generalizability of the findings, though the rich descriptions provide opportunities to understand the students’ experiences of the program designs. While both groups discussed the online program, most of the simulation students had limited to no experience of the online materials. While the survey responses aligned, the very small number of students that participated in the online program-specific focus group provide only a preliminary perspective of student views of this learning package. The differences in the ages and cultural diversity of the students in each program, as well as the over-representation of occupational therapy students, themselves provided insights into the diversity of learning needs and how the different program features met those. As a result, however, some groups were under-represented in aspects of this study, and it is not clear how they may have experienced the alternatives. Conducting the focus groups 10–14 days into the students’ subsequent placements provided a compromise between proximity to the program participation for recall, and the ability of students to reflect on their needs and achievements in preparation for placement with the knowledge of the actual placement requirements. Collecting data at another time point may have resulted in a different emphasis in the findings.

## Conclusion and recommendations

Overall, both programs addressed preparation for clinical placement, with each modality proving more advantageous at meeting different student learning needs. Offering complementary programs in this fashion may provide for comprehensive and yet efficient education, with the possibility that students from different backgrounds could be appropriately informed to choose one or more modalities to meet their personal needs based on their differing situations and histories. Through the in-depth investigation of students’ experiences, this study has revealed areas to optimize both simulation and online delivery. However, further research is required to confirm and extend these findings, and to investigate the continued outcomes for students beyond the program delivery with transferability into practice education.

The ability to apply learning and practice communication to build confidence were key in the simulation program being more advantageous in meeting the expectations of students selecting that program. The online program, on the other hand, was seen as accessible and useful, particularly for clinical documentation and clinical reasoning development. The importance of peer learning in both groups was also apparent in this study. The nature of the learning programs referenced in this study therefore exemplify ways in which educators can work to tailor preparation for similar foundational programs, including simulation, to maximize the learning of diverse students. The findings also support the continued targeted use of in-person simulation-based education for the application of learning prior to student practice education and placement experiences.

## Electronic supplementary material

Below is the link to the electronic supplementary material.

Supplementary Material 1

## Data Availability

The datasets used and/or analysed during the current study are available from the corresponding author on reasonable request.
