# Supplementary material for: Preparing allied health students for placement: a contrast of learning modalities for foundational skill development
Source: BMC Med Educ. 2023 Mar 15;23:161. doi: 10.1186/s12909-023-04086-7 (PMC10018923; doi:10.1186/s12909-023-04086-7)
Supplement: Supplementary file 1 — Supplementary Material 1 [file 12909_2023_4086_MOESM1_ESM.docx]

**Additional File 1: Pre-post questionnaire**

SD = Strongly Disagree   D = Disagree   U = Undecided   A = Agree   SA = Strongly Agree

1. I feel confident to establish rapport with a patient.
2. I feel confident to explain my professional role to a patient.
3. I feel confident to communicate empathetically and effectively with a patient using interpersonal skills such as reflective listening and appropriate use of questions.
4. I feel confident to adapt my communication style appropriately recognising the cultural and language diversity.
5. I feel confident to design and conduct an assessment safely.
6. I feel confident to identify key problem(s) during an assessment.
7. I feel confident to explain the problem(s) to a patient.
8. I feel confident to discuss the management plan with a patient/ carer/ relative.
9. I feel confident to select appropriate physiotherapy/ podiatry / occupational therapy interventions.
10. I feel confident to implement interventions safely and effectively.
11. I feel confident to provide information and education to patients.
12. I feel confident to complete written documentation to legal requirements.
13. I feel confident to use appropriate manual handling with patients.
14. I feel confident to practice appropriate infection control measures.
15. I feel confident to interact in a professional manner.
16. I feel prepared for placement.
17. I feel confident I can recognise my limitations or areas of weakness where I would benefit from further preparation for clinical placement.
